# Carnosic Acid Ameliorates Indomethacin-Induced Gastric Ulceration in Rats by Alleviating Oxidative Stress and Inflammation

**DOI:** 10.3390/biomedicines11030829

**Published:** 2023-03-09

**Authors:** Betul Danisman, Betul Cicek, Serkan Yildirim, Ismail Bolat, Deniz Kantar, Kirill S. Golokhvast, Dragana Nikitovic, Aristidis Tsatsakis, Ali Taghizadehghalehjoughi

**Affiliations:** 1 Department of Biophysics, Faculty of Medicine, Ataturk University, Erzurum 25240, Turkey; 2 Department of Physiology, Faculty of Medicine, Erzincan Binali Yildirim University, Erzincan 24100, Turkey; 3 Department of Pathology, Faculty of Veterinary, Atatürk University, Erzurum 25240, Turkey; 4 Department of Biophysics, Faculty of Medicine, Akdeniz University, Antalya 07058, Turkey; 5 Siberian Federal Scientific Centre of Agrobiotechnology, Centralnaya, Presidium, Krasnoobsk 633501, Russia; 6 Laboratory of Histology-Embryology, Medical School, University of Crete, 71003 Heraklion, Greece; 7 Department of Forensic Sciences and Toxicology, Faculty of Medicine, University of Crete, 71003 Heraklion, Greece; 8 Department of Medical Pharmacology, Faculty of Medicine, Bilecik Seyh Edebali University, Bilecik 11000, Turkey

**Keywords:** indomethacin, gastric ulcer, carnosic acid, inflammation, oxidative stress

## Abstract

Nonsteroidal anti-inflammatory drugs (NSAIDs) such as aspirin and indomethacin (IND) are the most commonly prescribed for inflammation or pain. However, widespread use causes several adverse effects, such as gastric ulcers, upper gastric system bleeding, and erosions. Carnosic acid (CA) is an important natural antioxidant found in rosemary (Rosmarinus essentials) and exhibits a protective effect by suppressing oxidative stress and inflammation. This study aimed to investigate the impact of CA on IND-induced gastric ulceration. Wistar male rats received CA (100 mg/kg) or esomeprazole (ESP) (20 mg/kg, standard drug) by oral gavage for 14 days, after that gastric ulceration was induced by oral administration of 100 mg/kg IND. CA pretreatment attenuated both gross morphological lesions and histopathological alterations. CA strongly reduced IND-induced oxidative stress, verified by a decrease in MDA (*p* < 0.001) and TOS levels (*p* < 0.05). Furthermore, an IND-dependent increase in CAT (*p* < 0.001) and GPx (*p* < 0.01) activities, as well as a reduction in GSH levels (*p* < 0.01), were ameliorated by CA pretreatment. CA also attenuated inflammatory damage by suppressing IL-1β (*p* < 0.01), IL-6 (*p* < 0.01), and TNFα (*p* < 0.001) production and increasing Nrf2/HO-1 (*p* < 0.05) expressions. In conclusion, CA shows a gastroprotective effect by reducing oxidative stress and attenuating inflammation.

## 1. Introduction

Non-steroidal anti-inflammatory drugs (NSAIDs) are widely prescribed for treating pain, fever, and inflammation. However, long-term use of the NSAIDs, such as indomethacin (IND) or aspirin, can cause gastric ulceration by various mechanisms, including injury through inhibition of prostaglandin (PG) synthesis, reduction in local blood flow, regional irritation, and inhibition of tissue regeneration [1,2]. The pathogenesis of gastric ulcerative lesion formation is multifactorial and has not been fully clarified. Even though several synthetic anti-ulcerative drugs are currently available, they can exhibit mild to severe side effects [3]. For example, omeprazole, a proton-pump inhibitor (PPI) that blocks the release of gastric acid, may facilitate *Clostridium* difficile infection [4], induce hypomagnesemia [5], or diminish anticoagulant drug efficiencies such as that of clopidogrel [6]. However, other PPIs, including ilaprazole, were shown not to disturb clopidogrel metabolism [7]. Another PPI, ranitidine, widely utilized for the treatment of gastroesophageal reflux and peptic ulcer disease, is indicated as a probable human carcinogen for some cancer types [8]. Indeed, studies suggest that ranitidine degradation results in the formation of high levels of a carcinogen, N-nitroso dimethylamine [9]. Therefore, it is necessary to identify non-toxic, easily accessible anti-ulcerative drugs [10]. 

Many studies have shown that gastric injury may occur due to increased reactive oxygen species (ROS) release, attenuated cell proliferation, and enhanced inflammation [11,12,13]. Therefore, controlling ROS generation and anti-inflammatory response is essential for abrogating gastric ulceration. 

Various plant-derived natural substances are effective in treating human disease. Rosmarinus officinalis L. is a popular culinary plant used in different parts of the world, exhibiting anti-inflammatory, antioxidant, antiangiogenic, and even anticancer effects. Rosmarinus officinalis L activity has been associated with various mechanisms, such as increased gene expression involved in modifying the immune response and specific metabolic pathways [14]. Carnosic acid (Salvin), structurally a phenolic diterpene, was initially isolated from Rosmarinus officinalis L. and Salva Officinalis L. leaves [15]. Notably, CA content depends on cultivating conditions. It is increased when plants are stressed, e.g., during drought [16]. To this diterpene, antioxidant and antimicrobial properties have been attributed, resulting in its examination for medical application in several pathologies [17,18]. Since the link between oxidative stress and gastric ulceration is well established, CA-acid, due to its antioxidative properties, is a plausible medication candidate. 

Previous studies have determined that CA exhibits higher antioxidant activity compared to commonly used synthetic antioxidants [19,20]. CA was found to scavenge ROS produced in the chloroplasts in plants, resulting in the synthesis of diterpene alcohols, primarily isorosmanol [21]. Furthermore, it has been suggested that CA removes ROS from plant membranes, facilitating their stability and protecting cell homeostasis [21,22,23].

Various studies have emphasized that CA-rich plant extracts have an anti-inflammatory effect by suppressing the release of inflammatory cytokines, including interleukin (IL)-1β, TNFα, and IL-6 in several cell types, including macrophages, but also in animal models [24,25,26,27,28]. Moreover, CA attenuates TNF-α downstream signaling by downregulating the inhibitor of nuclear factor κ-B (NF-κB) in combination with the enhancement of HO-1 expression [29]. Furthermore, due to augmenting the activity of the erythroid-derived 2-related factor 2 (Nrf2) transcriptional factor, CA attenuates TNF-α and nitric oxide (NO) inflammatory response [30]. 

**Figure 1:**
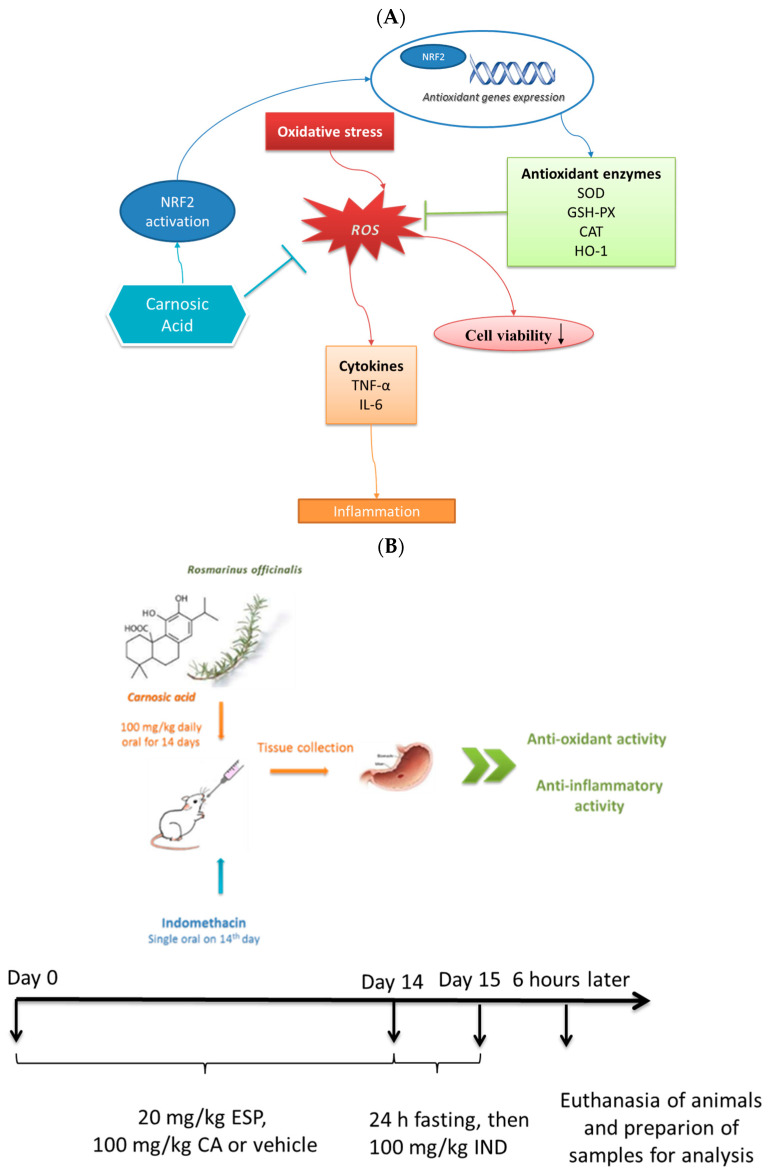
(**A**) Putative Mechanism of CA Action. (**B**) Diagram showing the design and time-course of experimental procedures (ESP, Esomeprazole at 20 mg/kg; vehicle, distilled water (98%); CA, Carnosic acid at 100 mg/kg; IND, Indomethacin at 100 mg/kg). All drugs were administrated by oral gavage. Data are reported as seven animals per group.

Notably, Nrf2 is a key transcription factor in regulating the cellular antioxidant response. Thus, Nrf2 downstream cascades are the primary protection mechanism that inactivates oxidative stress [31]. Indeed, the expression of genes encoding proteins involved in the antioxidant defense system and cytoprotective such as HO-1 are partly regulated by the Nrf2. ROS can oxidize the lipid and protein components of the cell, damaging the gastrointestinal tract barrier and thus enhancing gut permeability resulting in an inflammatory response [32]. Therefore, antioxidant enzymes like CAT or GPx, have a significant role against oxidative stress-induced ulcers. A possible mechanism of CA action is depicted in Figure 1A.

In the present study, we aimed to clarify the effects of CA in an IND-induced gastric ulcer model by investigating macroscopic, microscopic, and biochemical parameters. Thus, we examined the impact of CA on IND-induced TAS, TOS, IL1β, IL-6, TNF-α, Nrf-2, HO-1, MDA, and GSH levels as well on CAT and GPX activities and gastric lesions in gastric tissues.

## 2. Materials and Methods

### 2.1. Chemicals and Reagents

*Carnosic acid* (Sigma-Aldrich International, Darmstadt, Germany). Indomethacin (Endol 25 mg; 25 cap., DEVA Holding A.S., Istanbul, Turkey); Esomeprazole (Nexium 40 mg; 28 tablets, AstraZeneca Pharmaceutical Company, Istanbul, Turkey) were obtained. ELISA kits measuring IL-1β, IL-6, and TNF-α were obtained from Elabscience, TX, USA. TOS and TAC assays were purchased from Rel Assay Diagnostics, Gaziantep, Turkey. Hematoxylin Eosin (H&E) was obtained from (Merck, Darmstadt, Germany). The following antibodies were utilized: Nrf2 (Anti-Nrf2 antibody, Abcam, Boston, MA, USA, Catalog #: ab31163), HO-1 (Anti-HO-1 antibody, Abcam, Boston, MA, USA, Catalog #: ab13243). Fluorescein-5-Isothiocyanate (FITC; Abcam, secondary antibody, Boston, MA, USA, Catalog #: ab6785) and Texas Red (secondary antibody, Abcam, Boston, MA, USA, Catalog #: ab6719).

### 2.2. Animals

In this study, twenty-eight adult male albino Sprague-Dawley rats weighing between 250–300 g were used. The study was approved by Atatürk University Experimental Animal Ethics Committee with the number (E-42190979-000-2200190400). During the experiment, animals were fed rat chow and tap water ad libitum. The animals were housed in polypropylene cages, under a 12-h light/12-h dark regime, at 22 ± 0.5 °C and appropriate humidity. 

### 2.3. Experimental Design

The dosage of the tested agents was chosen according to the previous studies [33,34]. Rats were divided into four experimental groups each consisting of seven animals: control (no treatment was applied), IND-ulcerated animals (Gastric ulcer model, 100 mg/kg IND), IND + ESP (20 mg/kg ESP), IND + CA (100 mg/kg CA) [33,35]. CA, IND and ESP were dissolved in saline with 5% NaOH. CA (100 mg/kg CA) and ESP (20 mg/kg of ESP) were administered by oral gavage daily for 14 days. The control and IND (100 mg/kg IND) groups were given saline in the same way and volume. Gastric ulcer was induced on day 14 of the experiment by administering 100 mg/kg IND to animals of all groups except the control as summarized in Figure 1.

### 2.4. Generation of Gastric Ulcer Model

The gastric ulcer model was induced as described previously [36]. Briefly, all animals fasted 24 h before drug administration. Except for the control group, ulcers were induced by administering IND to the three experimental study groups, namely IND, IND+ ESP and IND + CA. The same volume of physiological saline was administered to the experimental animals as to the control group. 50 mg/kg ketamine and 5 mg/kg xylazine were administered to rats 6 h after IND administration. Anesthetized rats were euthanized by cervical dislocation, after which tissue samples were collected. Specifically, the stomach was opened along the greater curvature and washed with physiological saline at 4 °C. Washed stomach tissues were stored in tubes containing 10% formalin for histological procedures and at −800 °C for biochemical determination until analyses. Hematoxylin-eosin staining of the taken tissues was evaluated histopathologically and immunohistochemically.

### 2.5. Macroscopic Examination

The macroscopically examined gastric tissues were opened with the help of scissors, the mucosal layers were examined, and macroscopic images of the stomach tissues were taken. The gastric ulcer index was determined by utilizing the ToupView, Olympus program [12].

### 2.6. Ulcer Index and Preventive Index

The ulcer index and preventive index measurement were calculated using the method described by ElAshmawy et al. (2016) [12]. The ulcer score for each group was the mean number of ulcers in each group (total number of ulcers divided by the total number of rats), n = 7. The preventive index was calculated as the following: ulcer index of the ulcerated group − ulcer index of treated group × 100)/ulcer index of the ulcerated group. 

### 2.7. Histopathology

Tissue samples collected at the end of the evaluation were fixed in 10% formaldehyde solution for 48 h and embedded in paraffin blocks at the end of routine tissue follow-up procedures. Sections of 4 μm thickness were taken from each block, and the preparations destined for histopathological examination were stained with hematoxylin-eosin (HE) and examined with a light microscope (Olympus BX 51, JAPAN). A semiquantitative scoring system was used for histopathological evaluation as follows: -, no staining; +, mild staining; ++, moderate staining; +++, strong staining [37].

### 2.8. Immunohistochemical Analyse

Tissue sections taken on adhesive (poly-L-Lysin) slides for immunoperoxidase analysis were deparaffinized and dehydrated. Then, endogenous peroxidase was inactivated by incubating the sections in 3% H_2_O_2_ for 10 min. In continuation, the tissues were boiled in 1% antigen retrieval (citrate buffer (pH + 6.1) 100) solution and allowed to cool at room temperature. Sections were incubated with protein block for 5 min to prevent nonspecific background staining in tissues. Then, the primary antibody (IL33, Cat No: orb6205, Dilution Ratio: 1/100, UK) was applied to the tissues and incubated in accordance with the manufacturer’s instructions. 3-3′ Diaminobenzidine (DAB) chromogen was used for the development of the color stain. The stained sections were examined with a light microscope (Zeiss AXIO GERMANY).

### 2.9. Double-Immunofluorescence Assays

Tissue sections prepared on adhesive (poly-L-Lysin) slides for immunoperoxidase analysis were deparaffinized and dehydrated. Then, endogenous peroxidase was inactivated by keeping it in 3% H_2_O_2_ for 10 min. Subsequently, the tissues were boiled in 1% antigen retrieval (citrate buffer (pH + 6.1) 100) solution and allowed to cool at room temperature. Sections were incubated with protein block for 5 min to prevent nonspecific background staining. In continuation, the primary antibody (Nrf-2 Cat No: ab89443, Dilution Ratio: 1/100, UK) was applied to the tissues and incubated per the manufacturer’s instructions. Immunofluorescence secondary antibody was used as a secondary marker (FITC Cat No: ab6785 Diluent Ratio: 1/1000) and kept in the dark for 45 min. Subsequently, the second primary antibody (HO-1 Cat No: ab189491, Dilution Ratio: 1/100, UK) was dripped onto the tissues and incubated following the manufacturer’s instructions. A secondary immunofluorescence antibody was used as a secondary marker (Texas Red Cat No: ab6719 Diluent Ratio: 1/1000 UK) and kept in the dark for 45 min. In continuation, DAPI with mounting medium (Cat no: D1306 Dilution Ratio: 1/200 UK) was dripped onto the sections and kept in the dark for 5 min, and the sections were covered with a coverslip. The stained sections were examined under a fluorescent microscope (Zeiss AXIO GERMANY).

### 2.10. Determination of Oxidative Markers and Antioxidant Enzyme

Gastric tissues were homogenized in ice-cold PBS and centrifuged at 3000× *g* for 10 min. The supernatant was collected for measurement of catalase (CAT), glutathione peroxidase (GPx), glutathione (GSH), malondialdehyde (MDA) and Total Antioxidant Status (TAS)-Total Oxidant Status (TOS) levels. Protein quantification was performed using a BCA Protein Assay Kit (Pierce, Rockford, IL, USA). The levels of GSH, and lipid peroxidation levels (malondialdehyde (MDA) as well as GPx and SOD activities were measured using commercial kits according to the manufacturer’s instructions (Elabsicience, Nanjing, China). The TAS-TOS were quantified based on the manufacturer’s guidelines by using commercial kits [38,39].

### 2.11. Determination of Inflammatory Markers and Cytokines

IL-1β, IL-6, and TNF-α cytokine levels were measured in gastric tissue using a rat ELISA kit, as instructed. Briefly, samples were incubated at 37 °C for 90 min after addition to the plates. The wells were then emptied and biotinylated detection Ab working solutions were added and incubated for an additional 60 min. The plate was washed and incubated at 37 °C for 30 min by adding horseradish peroxidase conjugate working solution. Finally, substrate reagent and stop solution were added, respectively, and the plate was read at 570 nm using the Multiskan™ GO Microplate Spectrophotometer reader (Thermo Scientific, Waltham, MA, USA).

### 2.12. Statistical Analysis

SPSS 13.00 program was used for statistical analysis SPSS (SPSS for Windows, Inc., Chicago, IL, USA). The Shapiro–Wilk test for normality and the Levene test for homogeneity were performed. One-way analysis of variance (ANOVA) was used for the comparison of the groups in parametric conditions and the Tukey test was used for the post-hoc comparison. The nonparametric Kruskal–Wallis test was used for the analysis of the differences between the groups in the semiquantitative data obtained in the histopathological examination, and the Mann–Whitney U test was used for the comparison of the paired groups. In order to determine the intensity of positive staining from the pictures obtained as a result of immunohistochemical and double immunofluorescence staining, five random areas were selected from each image and evaluated in the ZEISS Zen Imaging Software program. Data were statistically defined and presented as mean and standard deviation (mean ± SD) for % area. *p* < 0.05 was considered significant.

## 3. Results

### 3.1. The Effects of CA on IND-Induced Gastric Tissue Macroscopic Alterations

**Figure 2:**
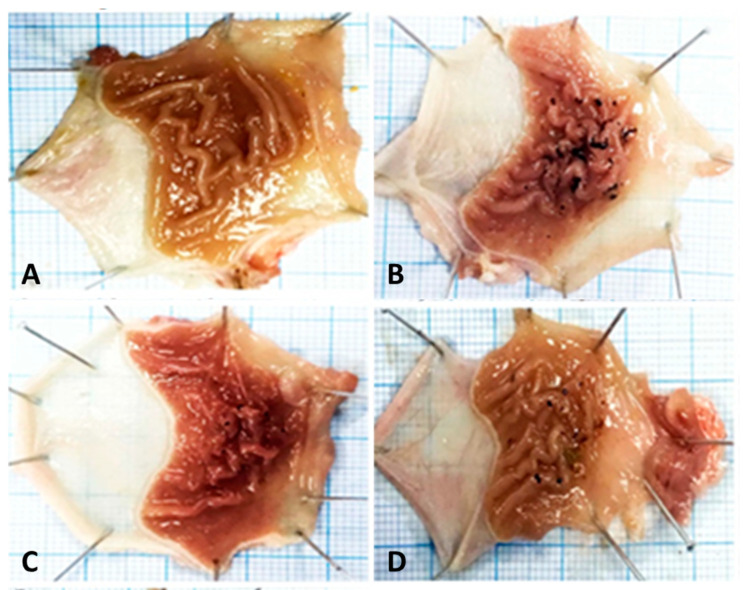
Photomicrograph showing the effect of CA on the gross morphology of the gastric mucosa of ulcerated rats. (**A**) Negative control group showed no injury of the gastric mucosa (**B**) IND- ulcerated rats (**C**) IND- treated rats pretreated with ESP (IND + ESP) (20 mg/kg) (**D**) IND- treated rats pretreated with (IND + CA) 100 mg/mL; IND: Indomethacin, ESP: Esomeprazole, CA: Carnosic acid.

The gastric tissues of the control animals exhibited normal gross morphology (Figure 2A) and Table 1. The IND-administered animals showed prominent mucosal folds and severe erosion; whereas pronounced ulceration and bleeding foci were observed in the gastric mucosa (Figure 2B) and Table 1. The macroscopic examination of ESP pretreated group (20 mg/kg) stomachs, revealed mild edema in the serosa, whereas mild erosion and bleeding foci in the gastric mucosa were observed. Based on macroscopic findings, the calculated ulcer score was significantly lower in the IND + ESP (*p <* 0.05) compared with the IND group (Figure 2C) and Table 1. These data verified the protective effect of ESP on gastric ulceration. Macroscopic gastric examination of CA (100 mg/kg) pretreated IND-administered animals revealed mild edema in the serosa, with mild parallel erosion of the mucosa. Furthermore, limited ulceration and bleeding foci were determined. Based on macroscopic findings, the calculated ulcer score was significantly lower in the IND + CA (*p* < 0.05) compared with the IND group (Figure 2D) and Table 1. Moreover, the protective effect of CA was equal to that of ESP (Figure 2) and Table 1.

The calculated Ulcer Score, Ulcer Index, and the effect of CA (Preventive Index) are presented in Table 1.

### 3.2. Histopathological Evaluation of CA Effects

**Figure 3:**
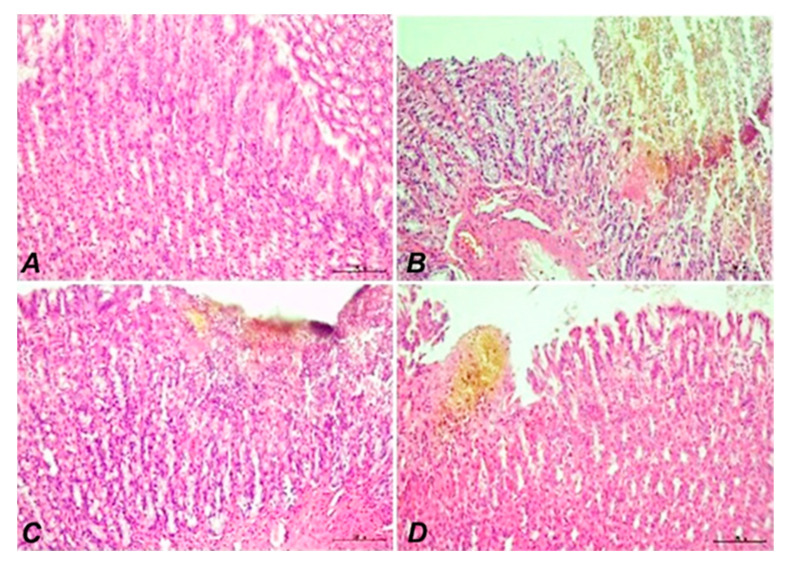
Photomicrographs showing the effect of CA on stomach sections of IND-administered rats stained with haematoxylin–eosin. (H&E) (**A**) Stomachs of negative control rats, (**B**) IND-administered rats (**C**), IND-administered rats pretreated with ESP (20 mg/mL) (IND + ESP) (**D**) and IND-administered rats pretreated with CA. IND: Indomethacin, ESP: Esomeprazole, CA: Carnosic acid. Bar: 100 µm.

The histopathological examination of hematoxylin and eosin-stained (H&E) gastric sections obtained from the control mice revealed normal histological structure (Figure 3A). Examination of the sections obtained from the IND group showed severe erosion of the mucosa, reaching down to the lamina muscularis. Furthermore, hemorrhagic infiltration, edema in the submucosa, and severe hyperemia of the vessels were observed (Figure 3B). Pretreatment with ESP (20 mg/kg) exhibited a significant protective effect on the tissue architecture. Thus, mild erosion in the mucosal layer, mild degeneration and necrosis of the mucosal epithelium, and mild hyperemia in the vessels were detected in the sections obtained from the ESP pretreated group (Figure 3C). The gastroprotective effect of CA was confirmed by histological analysis. Indeed, CA (100 mg/mL) exerted protective effects similar to ESP and partially ameliorated IND effects. Specifically, mild erosion of the mucosal layer, mild degeneration and some necrosis in the mucosal epithelium, and mild hyperemia in the vessels were detected (Figure 3D). The results of the histopathological evaluation are presented in Figure 3 and Table 2.

### 3.3. The Effect of CA on TNF-α Expression

**Figure 4:**
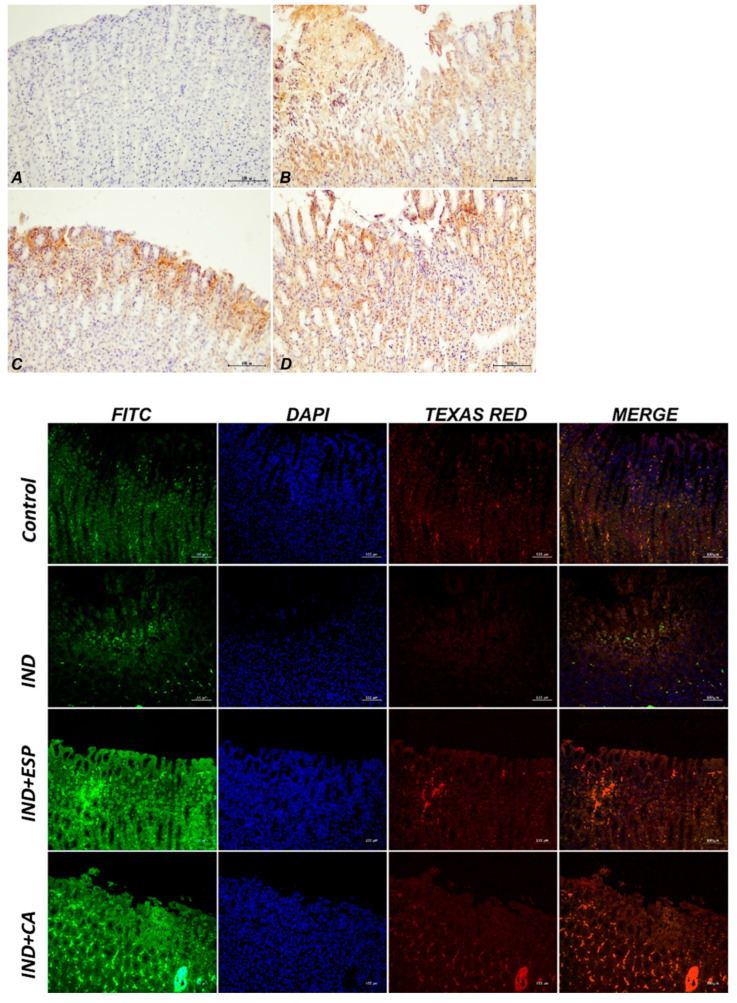
Gastric tissue, Nrf-2 expression (FITC), HO-1 Expression (Texas Red), D-IF, 4′,6-Diamidino-2-Phenylindole (DAPI), Fluorescein Isothiocyanate (FITC), IND: Indomethacin, ESP: Esomeprazole, CA: Carnosic acid. Bar: 100 µm.

In continuation, the expression of TNF-α in rat tissue sections was evaluated by immunohistochemistry and the results are presented in Figure 4 and Table 3. Gastric tissues of control rats were negative for Tnf-α expression (Figure 4A). Tissues collected from IND-administered animals showed intense Tnf-α expression, especially in areas of eroded gastric glands (arrow), interstitial space, surrounding vessels, and in the cytoplasm of invading inflammatory cells (Figure 4B). Gastric tissue from IND-administered rats pretreated with ESP showed moderate TNF-α reaction in the eroded areas, in the interstitial space and around the vessels (Figure 4C). Moderate Tnf-α immunoreactivity was detected in the eroded gastric glands, interstitial space and in the vicinity of vessels in gastric tissues collected from IND-administered rats pretreated with CA (Figure 4D). These data demonstrate that CA protects against adverse IND effects. The immunohistochemical findings are summarized in Table 3.

### 3.4. Biochemical Results

#### 3.4.1. Gastric Tissue Oxidant and Antioxidant Parameters 

To further clarify the effect of CA on IND-induced gastric ulceration MDA, TOS, GSH and TAS levels were measured and CAT and GPx activities were determined. As shown in Table 4 IND administration significantly increased MDA levels compared to the control animals (*p* < 0.0001). Notably, rats pre-treated with both ESP and CA exhibited significantly reduced MDA levels compared to just IND-administered rats, (*p* < 0.0001), respectively. Similar to MDA findings, TOS levels measured in the IND group were significantly higher compared to the control (*p* < 0.01). Pretreatment with ESP and CA caused a significant decrement in TOS levels (*p* < 0.01). IND treatment strongly reduced TAS levels compared with the control group. This decrease was significantly reverted by CA and ESP pretreatments (*p* < 0.05). These data show that CA attenuates oxidative insult similarly to ESP.

In line with these findings, IND administration caused a decrease in CAT and GPx activities and GSH levels, while pretreatment with ESP and CA reverted the downregulation. Specifically, the decrease in CAT and GPx activities in the IND group was attenuated in ESP and CA treatment groups, respectively, (*p* < 0.001 and *p* < 0.01). Likewise, the marked reduction of GSH levels in the IND group was ameliorated in IND + ESP and IND + CA groups (*p* < 0.01) (Table 4). 

#### 3.4.2. Inflammation Markers

As shown in Table 5, IND administration causes a significant increase in the expression of inflammatory markers such as IL-1β, IL-6 and TNF-α compared to the control group. Pretreatment with CA or ESP strongly attenuated the IND effect. 

## 4. Discussion

NSAIDs are widely used in the treatment of pain, fever, and inflammation. Indeed, NSAIDs administration due to their widespread usage and easy availability is one of the most common causes of gastric ulceration. IND, an NSAID, triggers ulcer formation by inhibiting prostaglandin production and causing excessive production of free oxygen radicals [40]. 

The current study evaluated the effect of CA in an IND-induced gastric ulceration model. The induction of gastric ulceration was executed by the oral administration of IND (100 mg/kg bw). Well in accordance with previous investigations, IND administration caused hemorrhagic macroscopic lesions and a high ulcer index. Furthermore, ulcerated animals exhibited typical histological changes including mucosal thickness decrease, erosion of the gastric glands, damage to the integrity of the gastric mucosa, submucosal edema, and inflammatory cell infiltration [41,42].

Pretreatment with CA exhibited a macroscopic gastroprotective effect which was confirmed by microscopic histopathological findings. Notably, the amelioration of the macroscopic ulcer scores by CA was found to be quite similar to ESP, a verified gastroprotective drug. 

ROS are produced during cell metabolism under both physiological and pathological processes [43]. However, excessive ROS release is exhibited in the disease [44,45]. Organisms defend against ROS adverse effects with specific antioxidant systems, composed of enzymes and endogenous scavengers. Indeed, CAT and SOD enzymes are characterized as the first line of defense against oxidative stress. SOD neutralizes superoxide and produces hydrogen peroxide (H2O2), and CAT eliminates H2O2 harmful effects by converting it into water [46]. When excessive ROS are released, these defense mechanisms are overwhelmed. IND was previously shown to decrease CAT activity [12,47], which is verified in the present study. Furthermore, here we show that exogenous CA treatment upregulated the CAT levels of IND-ulcerated animals. The observed increased CAT activity upon CA administration may be due to CA antioxidant effects or may be a direct effect on CAT expression. Indeed, previous studies showed that CA application increases CAT activity [48,49]. Increased CAT activity, will prevent lipid peroxidation and tissue destruction documented by attenuated IND-induced lesions in CA-pretreated animals [50].

As ROS production increases, the levels of scavengers e.g., GSH and other endogenous antioxidants, decrease, facilitating oxidative tissue damage [51]. The results of this study show that CA pretreatment of IND-administered animals increases their GSH levels similar to the control drug ESP, confirming CA scavenger properties. 

GPx catalyzes the reduction of both hydrogen peroxide and lipid peroxides and protects cellular proteins against pathological changes. However, in our study, IND-administered animals exhibited GPx depletion, which promotes ROS generation and oxidative stress generation. The final result of GPx failure is a dysregulation of the functional and structural integrity of cell and organelle membranes [52]. CA reverted GPx downregulation in IND-administered animals. In summary, pre-treatment with CA reduced MDA and TOS levels and strengthened the cellular antioxidant defense mechanism by increasing, CAT and GPx activities and GSH and finally, TAS levels.

Exogenous antioxidant intake supports the defense system by scavenging ROS and reducing oxidative damage. Most endogenous antioxidants are encoded by Nrf2-KEAP1 system [53]. It has been suggested that CA contributes to antioxidant activity by increasing Nrf2 expression in different tissues [54]. Thus, Yang et al. [33] show that administering CA in an ulcerative colitis model ameliorated oxidative stress by increasing Nrf2 and antioxidant enzyme expressions. Indeed, enhancing the Nrf2 pathway can potentially reduce oxidative damage and ulcer formation. 

The current study shows for the first time that CA increases Nrf2 and the Nrf2 downstream target HO-1 expressions and reduces oxidative damage in animals subjected to IND insult. 

The second aim of this study was to examine the effects of CA on the levels of pro-inflammatory markers in the gastric tissue of IND-induced rats. It is well established that in gastritis and peptic ulcer, a massive infiltration of activated neutrophils is evident, as well as an upregulation of pro-inflammatory cytokines release such as TNF-α and IL-1 [55,56]. Moreover, NSAIDs were shown to increase neutrophil IL-6 secretion in the damaged gastric mucosal tissues. Indeed, the migration of neutrophils to the damaged area is considered a precursor of damage [23,57].

Here, we show that CA exerts a potent anti-inflammatory effect as it attenuated IND-dependent increase of TNFα, IL1β, and IL6 expression in rat gastric tissues. The attenuation of pro-inflammatory factors was correlated with the preservation of gastric mucosa histological structure and with decreased oedema and inflammatory cells infiltration.

Our study presents several limitations. Firstly, one CA dose and treatment duration were examined. Therefore, dose dependency and the effective dose period of administration need to be established. Furthermore, even though animal models provide important input, extrapolation to humans requires further extensive study.

## 5. Conclusions

The aim of this study was to investigate the impact of CA on IND-induced gastric ulceration in rats. Pretreatment with exogenous CA suppressed both gross gastric morphological lesions and histopathological alterations. The effects were perpetrated through the modulation of oxidative stress and the immune response. Thus, CA pretreatment decreased oxidative stress by increasing CAT and GPx activities, upregulating GSH, and decreasing MDA. This resulted in the increase of TAS and attenuation of TOS. Furthermore, CA strongly downregulated the expression of inflammatory markers, including TNFα, IL1β, and IL6. In summary, CA exerts gastroprotective effects with significant therapeutical implications. Future studies need to focus on elucidating the specific aspects of the CA mechanism of action. As a limitation of study, CA protective effects on stomach tissue can be evaluated for COX, PH and stomach volume.

## Figures and Tables

**Table 1:** Effects of CA on IND-induced macroscopic gastric injuries. Ulcer Score, Ulcer Index and Effect of Carnosic acid (Preventive Index).

	Ulcer Score	Ulcer Index	Preventive Index
Control	-	-	-
IND	29.00 ± 0.86 ^a^	2380	-
IND + ESP	1.45 ± 0.76 ^b^	140	%91.39
IND + CA	1.84 ± 0.69 ^b^	170	%87.65

Data for ulcer scores are expressed as means ± SE (n = 7 rats/group), and letters in columns (a,b) show a statistical difference compared to the IND-group (*p* < 0.05).

**Table 2:** Effects of CA on IND-induced microscopic gastric injuries.

	Erosion	Ulceration	Oedema in the Serosa	Bleeding and Hyperemia
Conrtol	-	-	-	-
IND	+++	+++	+++	+++
IND + ESP	+	+	+	+
IND + CA	+	+	+	+

Scoring of histopathological findings observed in stomach tissues.

**Table 3:** Effects of CA on IND-induced TNF-α, Nrf-2 and HO-1 expressions.

	Tnf-α	Nrf-2	HO-1
Conrtol	20.27 ± 0.18 ^a^	44.85 ± 1.65 ^a^	40.12 ± 1.26 ^a^
IND	79.25 ± 2.92 ^b^	30.41 ± 0.77 ^b^	25.37 ± 0.49 ^b^
IND + ESP	39.22 ± 1.12 ^c^	83.71 ± 1.85 ^c^	77.19 ± 2.17 ^c^
IND + CA	38.93 ± 1.66 ^c^	80.97 ± 2.19 ^c^	75.6 ± 2.49 ^c^

a,b,c; Different letters on the same line represent a statistically significant difference. (*p* < 0.05).

**Table 4:** Effects of CA on the oxidant and antioxidant parameters in gastric tissues of ulcerated rats.

Groups/Parameters	Control	IND	IND + ESP	IND + CA
TAS (U/mg protein)	1.04 ± 0.03	0.66 ± 0.05 *	1.12 ± 0.12 #	0.78 ± 0.10 *,#
TOS (µmol H_2_O_2_ Equiv/L)	4.39 ± 0.33	5.66 ± 0.18 *	4.76 ± 1.90 #	4.50 ± 2.56 #
CAT (U/mg protein)	1110.73 ± 207	597.10 ± 91.57 ***	1062.21 ± 128.56 ###	1081.57 ± 139.27 ###
MDA (nmol/mg protein)	6.89 ± 0.96	21.32 ± 1.48 ***	9.12 ± 1.79 *,###	6.38 ± 0.09 ###
GSH (nmol/mg protein)	0.28 ± 0.04	0.13 ± 0.02 **	0.24 ± 0.03 ##	0.19 ± 0.03 *,#
GPx (U/mg protein)	103.26 ± 12.99	46.20 ± 12.23 **	73.18 ± 7.24 **,###	88.21 ± 10.93 **,###

Data are expressed as mean ± SEM (n = 7/group). * *p* < 0.05, ** *p* < 0.01, *** *p* < 0.001 versus control group, # *p* < 0.05, ## *p* < 0.01, ### *p* < 0.001 versus IND group. TAS: total antioxidant status; TOS: total oxidant status; CAT: catalase; GSH: glutathione; GPx: glutathione peroxidase; IND: indomethacin; ESP: esomeprazole; CA: carnosic acid. Data are expressed as mean ± SD.

**Table 5:** Effects of CA on inflammatory markers in gastric tissues of ulcerated rats. Data are expressed as ± SEM, n = 7.

Groups/Parameters	Control	IND	IND + ESP	IND + CA
IL-1β (nmol/mprotein)	26.08 ± 2.10	39.38 ± 1.74 **	26.50 ± 0.81 **,##	32.52 ± 1.22 **,##
TNFα (ng/L)	64.50 ± 10.41	163.7 ± 17.70 ***	103.3 ± 4.328 ***,###	152.1 ± 16.28 ***,###
IL-6 (U/mg protein)	21.78 ± 3.15	41.63 ± 3.24 **	29 ± 3.03 **,###	36.67 ± 2 **,##

** *p* < 0.01, *** *p* < 0.001 versus control group, ## *p* < 0.01, ### *p* < 0.001 versus IND group). IL-1β: interleukin 1 beta; TNFα: tumor necrosis factor alpha; IL-6: interleukin 6; IND: indomethacin; ESP: esomeprazole; CA: carnosic acid.

## Data Availability

We declare that all data supporting the findings of this study are available.
